# First Description of Reduced Pyruvate Dehydrogenase Enzyme Activity Following Subarachnoid Hemorrhage (SAH)

**DOI:** 10.3389/fnins.2017.00037

**Published:** 2017-02-14

**Authors:** Nadine Lilla, Hannah Füllgraf, Christian Stetter, Stefan Köhler, Ralf-Ingo Ernestus, Thomas Westermaier

**Affiliations:** Department of Neurosurgery, University of WürzburgWürzburg, Germany

**Keywords:** aerobic glycolysis, CBF, metabolism, PDH, SAH, secondary brain damage

## Abstract

**Object:** Several previous studies reported metabolic derangements and an accumulation of metabolic products in the early phase of experimental subarachnoid hemorrhage (SAH), which may contribute to secondary brain damage. This may be a result of deranged oxygen utilization due to enzymatic dysfunction in aerobic glucose metabolism. This study was performed to investigate, if pyruvate dehydrogenase enzyme (PDH) is affected in its activity giving further hints for a derangement of oxidative metabolism.

**Methods:** Eighteen male Sprague-Dawley rats were randomly assigned to one of two experimental groups (*n* = 9): (1) SAH induced by the endovascular filament model and (2) sham-operated controls. Mean arterial blood pressure (MABP), intracranial pressure (ICP), and local cerebral blood flow (LCBF; laser-Doppler flowmetry) were continuously monitored from 30 min before until 3 h after SAH. Thereafter, the animals were sacrificed and PDH activity was measured by ELISA.

**Results:** PDH activity was significantly reduced in animals subjected to SAH compared to controls.

**Conclusion:** The results of this study demonstrate for the first time a reduction of PDH activity following SAH, independent of supply of substrates and may be an independent factor contributing to a derangement of oxidative metabolism, failure of oxygen utilization, and secondary brain damage.

## Introduction

Subarachnoid hemorrhage (SAH) is a disease with very poor prognosis. Approximately 15% of patients die before they are admitted to hospital, the overall mortality is as high as 50%. These numbers illustrate the significance of secondary brain damage after SAH (van Gijn and Rinkel, 2001). A variety of harmful events like intracerebral hemorrhage, occlusive hydrocephalus, and a steep increase of ICP and decrease of cerebral perfusion pressure (CPP) cannot be prevented, because their onset is before hospital admission. However, these events are in the focus of emergency management when SAH is diagnosed. At the time, the diagnosis is established and treatment has to start, the phase of global perfusion disturbance is eased already in many patients as ICP has largely recovered after the initial steep increase (Westermaier et al., 2009a). However, various mechanisms of secondary brain damage, which have been reported to arise after SAH or after global brain ischemia, are potential targets for further therapy (Baethmann et al., 1991; Siesjö et al., 1995). Extra-/intracellular ion-shifts, an excessive glutamate release, an early formation of brain edema and acute vascular and molecular changes have been characterized as such pathophysiological mechanisms (Sehba and Bederson, 2006). All these mechanisms finally lead to an imbalance of metabolic demand and oxygen supply. Carpenter et al. showed by positron emission tomography (PET) in SAH patients on days 2–5 after aneurysm rupture a significant reduction of the cerebral metabolic rate of oxygen (CMRO_2_) of 25%, suggesting primary metabolic alterations and an uncoupling of cerebral blood flow (CBF) and metabolism after SAH (Carpenter et al., 1991). Information on metabolism in the first hours after SAH is rare although this period may be decisive for the final outcome after aneurysmal SAH since the neurological performance at the time of first evaluation is one of the best and most reliable prognostic factors. In previous studies, we found, that despite incomplete recovery of CBF for more than 6 h after experimental SAH, tissue oxygenation recovered to baseline level 2 h after SAH and significantly exceeded this level up to 140% and more of baseline level after 6 h (Westermaier et al., 2009b). Furthermore, a persistent increase of lactate and a secondary increasing level of pyruvate concentration after SAH was monitored (Westermaier et al., 2011). These observations suggest a persistent failure of aerobic glycolysis after SAH. The reason for this failure is unknown to date. The purpose of this study was to investigate, if the activity of pyruvate dehydrogenase enzyme (PDH)—as a key enzyme to the TCA cycle—is deranged in the early phase of experimental SAH.

## Materials and methods

For the experiments, 18 male Sprague-Dawley rats (body weight of 250–300 g) were used, provided by Harlan Winkelmann, Bochum, Germany. All experiments were approved by the regional ethics authorities on animal research and the district government of Bavaria, Germany, and in conformity with the German Law for animal protection and the National Institute of Health Guidelines for Care and Use of Laboratory Animals.

### Animal preparation and monitoring

Induction of anesthesia was performed with 4% isoflurane, followed by oral intubation and mechanical ventilation with an air-oxygen mixture to provide normal blood gases. After induction of anesthesia, isoflurane was lowered to 2.5% for surgical procedures and to 1.5% from 30 min before induction of SAH until the end of monitoring period. Temperature was constantly measured throughout the whole experiment, maintaining temperatures at 37°C. Continuous arterial blood pressure measurement was performed by cannulating the tail artery. Blood gas samples were taken 30 and 5 min before SAH, 30 min after SAH and hourly until end of experiment.

### Laser doppler flowmetry and intracranial pressure

After induction of anesthesia and oral intubation, a midline incision of the scalp was performed and two burr holes, 1 mm dorsal and 5 mm lateral of the bregma, were drilled without injuring the underlying dura mater. For continuous bilateral monitoring of local cerebral blood flow (LCBF), a two-channel laser-Doppler flowmeter (MBF3 Laser-Doppler Flowmeter [LDF], Moor Instruments, Axminster, England) was used and data was digitally recorded with a frequency of 2 Hz.

For continuous measurement of ICP an additional burr hole was drilled 3 mm lateral and 0.5 mm anterior of the bregma over the left frontal cortex.

Thereafter, animals were put in supine position and fixed in a stereotactic frame. With the help of a micromanipulator, the rectangularly bent LDF-probes (P5bs, Moor Instruments) were positioned in each burr hole in the area of the cortex supplied by the middle cerebral artery (MCA). Also with the help of a micromanipulator the intraparenchymal ICP probe (Camino, Integra Neurosciences, Plainsboro, NJ, USA) was inserted with a depth of 2 mm into the brain.

### Induction of SAH

SAH was induced by the endovascular puncture model (Bederson et al., 1995; Veelken et al., 1995). After a right paramedian longitudinal incision, the external carotid artery was ligated and exiting branches were coagulated. Afterwards, temporary aneurysm clips were put on the common and internal carotid arteries. The external carotid artery was incised about 6 mm distal the carotid bifurcation and a 3.0 Prolene filament (Ethicon, Inc. Somerville, New Jersey) was inserted and secured with a silk ligature. Then temporary clips were removed, and the external carotid artery was cut. Thus, the filament could be moved intracranial via the internal carotid artery. After advancing approximately 25 mm, a clear descent of ipsilateral CBF was seen, indicating a correct position of the filament in the internal carotid artery in front of the exit of the MCA. The filament was then advanced 3 mm further perforating the vessel in the area of the anterior cerebral artery (ACA). Immediately thereafter, the filament was quickly pulled back into the external carotid artery ascertaining reperfusion of the internal carotid artery. SAH was verified by a sharp bilateral decrease of LCBF and a sudden increase of ICP. Continuous measurement of MABP, ICP and CBF parameters was performed for 3 h after vessel perforation. After 3 h, the animals were decapitated. Brains were removed, dissected on a cooling plate and immediately put into fluid nitrogen (after approximately 60 s) after being stored at −80°C for further experiments.

### Sample preparation and enzyme linked immunosorbant assay (ELISA)

Brain samples of the right frontal hemisphere were defrosted and homogenized in phosphate buffered saline (PBS) and protein concentrations determined by the Bradford method. Protein concentrations ranged around 27.40 ± 4.18 mg/ml. Thereafter, PDH activity was measured by the PDH Enzyme Activity Microplate Assay Kit (MitoScience, Eugene, Oregon, USA. ELISA measurement was performed using an ELISA Reader (Multiskan EX, Labsystems, Finnland). Kinetic measurement was performed by an optical density (OD) of 450 nm every 10 s up to 3 min with interphase mixing using the kinetic software GENESIS (Genesis Lite, Version 2.16, Life Sciences, UK).

### Experimental groups

The rats were randomly assigned to one of two groups (*n* = 9): (1) SAH induced by the endovascular filament model and (2) sham operated controls. In the sham group, a 3.0-prolene filament was also inserted into the external carotid artery, moved further via the internal carotid artery and was then withdrawn again without perforating a vessel.

### Statistical analysis

Statistical analysis was performed with GraphPad Prism 4 Statistical Software (GraphPad, San Diego, California, USA). Physiological data of each point in time as well as ICP and LDF were analyzed using the unpaired student's *t*-test. A *P* < 0.05 was considered to be significant. Results are presented as mean ± standard deviation.

To test the hypothesized differential development of enzyme activity between control and SAH animals for statistical difference we calculated a regression slope for each brain and compared the slopes by Mann-Whitney U-tests.

## Results

### Physiological parameters

There were no statistically significant differences between SAH and sham operated group regarding pH, pO_2_, and pCO_2_ (data not shown).

### Intracranial pressure (ICP), mean arterial blood pressure (MABP) and cerebral perfusion pressure (CPP)

**Figure 1:**
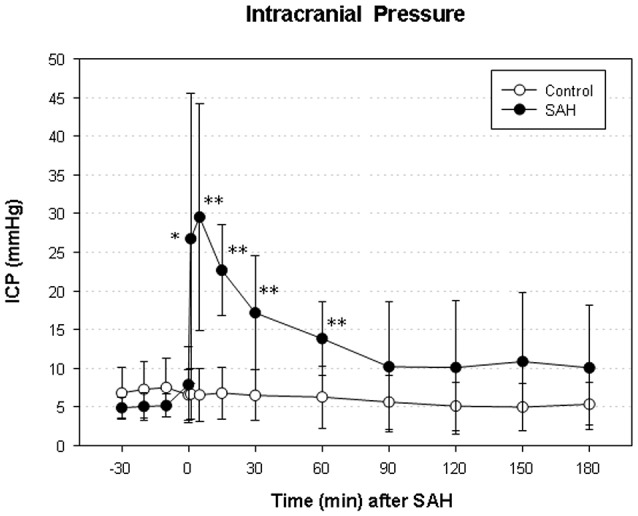
**Time course of intracranial pressure (ICP) in subarachnoid hemorrhage (SAH) group and sham-operated group**. Measurement from 30 min before until 180 min after induction of SAH. Values are presented as mean ± standard deviation. ^*^p < 0.05 and ^**^p < 0.01 vs. control group in each time point.

**Figure 2:**
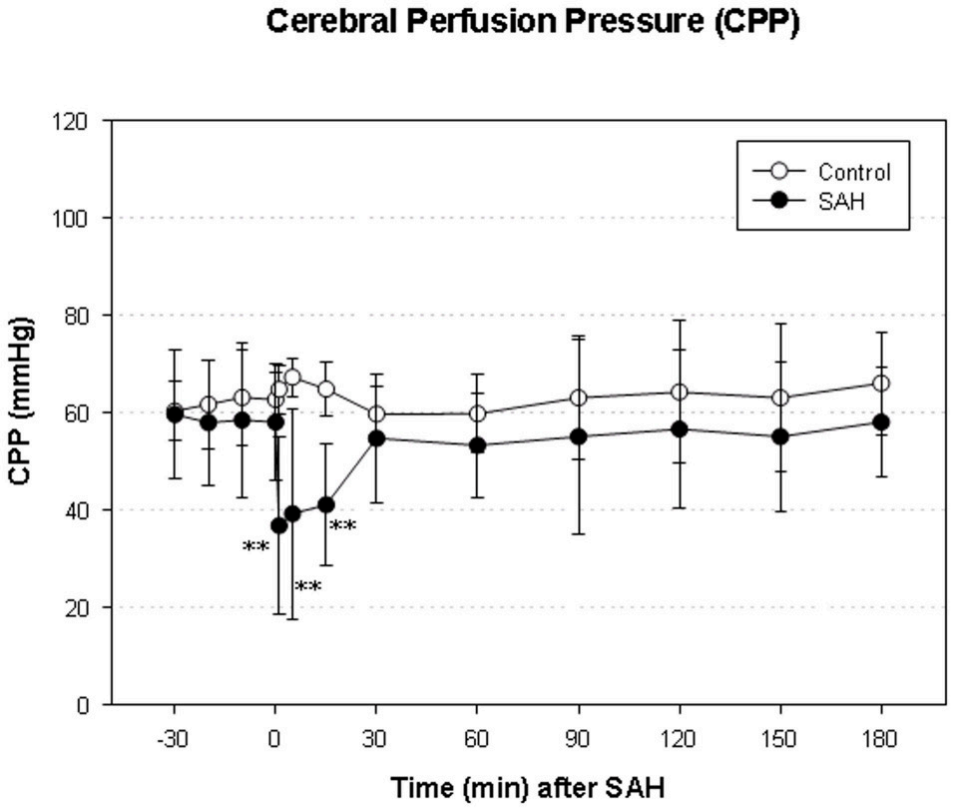
**Time course of CPP in SAH group and sham-operated group**. Measurement from 30 min before until 180 min after induction of SAH. Values are presented as mean ± standard deviation. ^**^p < 0.01 vs. control group in each time point. The initial increase ICP is combined with an immediate decrease of CPP—significantly reduced in the SAH group until 15 min after SAH.

We did not find a significant elevation of MABP in the present series (data not shown). The maximum elevation of ICP was 30 ± 14 mmHg 5 min after vessel perforation in animals subjected to SAH. CPP decreased from a baseline of 58 ± 12 mmHg to a minimum of 37 ± 18 mmHg and almost completely recovered after 30 min. The courses of ICP and CPP are depicted in Figures 1, 2.

### Local cortical blood flow (LCBF)

**Figure 3:**
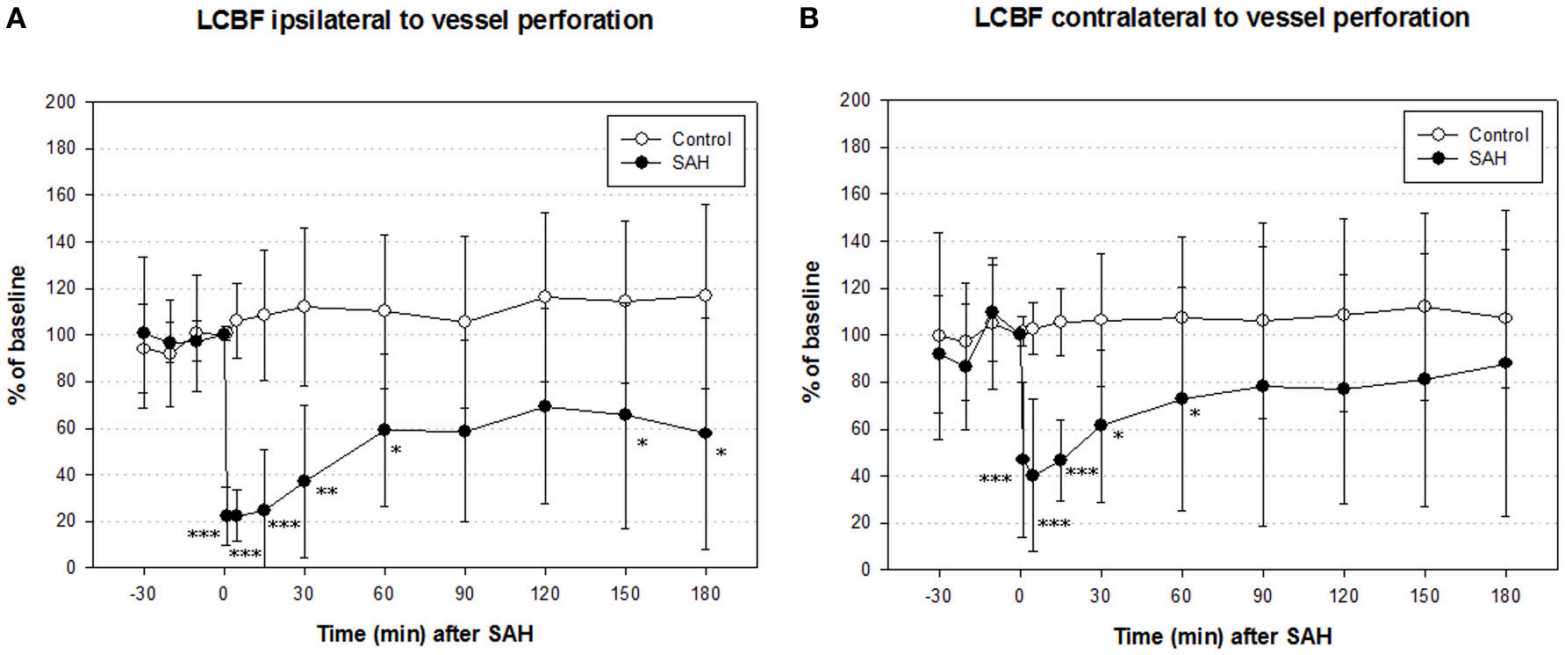
**Time course of local cerebral blood flow (LCBF) ipsilateral (A)** and contralateral **(B)** of vessel perforation/sham operation in SAH group and sham-operated controls. Measurement from 30 min before until 180 min after induction of SAH. Values are presented as mean ± standard deviation. ^*^p < 0.05, ^**^p < 0.01, and ^***^p < 0.001 vs. control group in each time point. The significant decrease of LCBF exceeded the decrease of CPP immediately after SAH until the end of measurement.

In the SAH group, ipsilateral LCBF declined to a minimum of 22 ± 12% of baseline 1 min after SAH, recovered to 59 ± 32% after 1 h and picked up further to 66 ± 48% at the end of measurement. In the control group, ipsilateral LCBF slightly increased to 110 ± 43% of baseline and showed no significant changes throughout the whole experiment. The decline of ipsilateral LCBF in the SAH group was significant from induction of SAH until the end of monitoring period 3 h after SAH (Figure 3 left).

Contralateral LCBF in SAH group declined to a minimum of 40 ± 32% of baseline 5 min after induction of SAH, recovered to 73 ± 48% after 1 h and further to 98 ± 65% after 3 h at the end of experiment. In the sham group, contralateral LCBF slightly increased to 108 ± 35% of baseline after 1 h and did not show any significant changes throughout the whole experiment as well. The decline of contralateral LCBF in SAH group was significant from induction of SAH until 60 min after SAH (Figure 3 right).

### Pyruvate dehydrogenase enzyme activity

**Figure 4:**
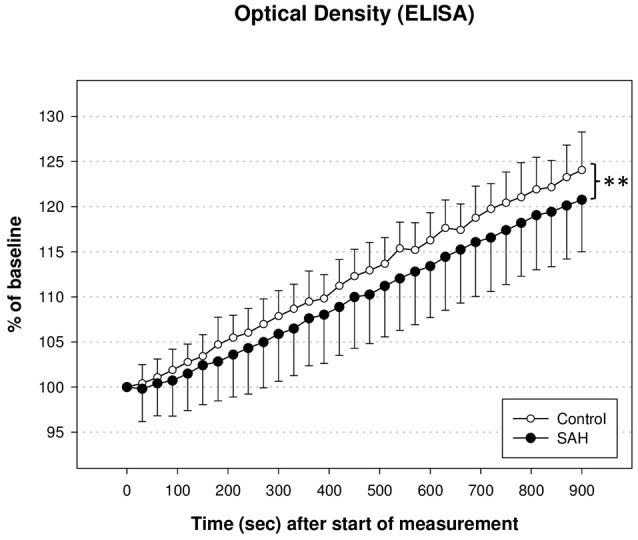
**Activity of pyruvate dehydrogenase enzyme (PDH) measured via enzyme linked immunosorbant assay (ELISA) and illustrated as optical density in % of baseline**. PDH activity in SAH animals differed significantly from PDH activity in control animals (^**^p < 0.01).

PDH activity measured via ELISA and illustrated as optical density in % of baseline at 450 nm showed a decent trend of reduction during the whole time course of evaluation. As shown in Figure 4, PDH activity in SAH animals differed significantly from PDH activity in control animals (U_1_ = 410; U_2_ = 162; IzI = 123,629 and therefore *p* < 0.01). There was no significant correlation between the reduction of PDH activity and the reduction of CBF in either hemisphere 3 h after induction of SAH, nor any time-point before.

## Discussion

Although the physiological changes in this present series were rather moderate compared to previous reports using the same experimental model (Prunell et al., 2004; Westermaier et al., 2009b, 2011) this study shows a clear depression of PDH activity in rats subjected to SAH. To our knowledge, there has been no comparable study reported in literature so far, investigating the PDH activity in the first hours after SAH in humans nor in experimental models. The reduced activity of PDH after SAH, combined with previous findings of elevated glutamate and lactate levels, secondary and persistent elevation of pyruvate levels, and a significant increase of the lactate-pyruvate ratio (LPR) (Westermaier et al., 2009b, 2011) suggest a derangement of oxidative metabolism in the early phase of SAH. In previous studies using the same experimental model, we found no signs of cerebral infarction or hypoxic brain damage (Westermaier et al., 2009a,b, 2011). Instead, we observed selective damage in hippocampal neurons. This is in accordance with our finding that CBF rather quickly recovers to values above ischemic thresholds as previously defined by various authors (Jones et al., 1981). It is one limitation of this experimental model that the extent of SAH and of pathophysiological changes is characterized by a larger variability (Prunell et al., 2004). Thus, mean values of CBF presented in Figure 3 must be interpreted with caution since they do not exclude that some animals may have been in a CBF-range where functional metabolism could already be deranged. The reduction of PDH activity in SAH animals, however, was highly consistent and significant suggesting that its reduction after SAH is not linearly correlated to CBF.

Our observation that the reduction of PDH activity was independent of the reduction of CBF suggests that there might be a primary deficiency of the PDH complex in the early stage of SAH.

### Pyruvate dehydrogenase enzyme and complex

Pyruvate dehydrogenase enzyme—located in the outer mitochondrial matrix—is a key enzyme to the TCA cycle and oxidative phosphorylation and, therefore, a critical link between anaerobic and aerobic metabolism (Reed, 1981). Several studies of PDH function and activity have been conducted in experimental models of cerebral ischemia and traumatic brain injury (TBI), but not in SAH so far. Since the pathophysiology of SAH is different from stroke (a focal ischemia) and post-TBI (a complex multifocal ischemia) it is to be understood as a completely different and discrete disease pattern. In contrast to ischemia (in stroke and TBI), the sudden and distinct decrease of CBF after SAH is not followed by an appropriate reperfusion, but recovers slowly and incompletely—rather like a hypoperfusion persisting for several hours (Westermaier et al., 2009b).

In experimental rat brain ischemia, Zaidan and Sims (1997) demonstrated a reduced activity of PDH in the dorsolateral striatum after 30 min of forebrain ischemia—particularly in selectively vulnerable neurons. Furthermore, it was shown, that the reduction of PDH activity in the frontal cortex was reperfusion-dependent (Bogaert et al., 1994). Because there was no change of PDH activity in ischemia alone, the authors suggested that the reduction of PDH activity was a factor of reperfusion injury.

Regarding reperfusion injury, there might be a difference compared to SAH. It was shown in this and previous experimental SAH rat model studies, that CPP decreases significantly after induction of SAH, but recovers quickly and is almost restored to normal values after 30 min. LCBF, in turn, also immediately decreases after induction of SAH, but recovers much slower and is still markedly reduced after 6 h. LCBF therefor clearly exceeds the decline of CPP in its duration and extent. This resembles a kind of a “low flow status” in the early hours after SAH. The persistent hypoperfusion might lead to an exceeding and/or longer lasting derangement of oxidative metabolism, as neurons quickly shift from aerobic to anaerobic metabolism once the LCBF is lower than 1/3 of normal values (Siesjo, 1992). This shift from aerobic to anaerobic metabolism leads to an increase of pyruvate (Westermaier et al., 2011)—and to changes in the ATP/ADP-ratio, the NADH/NAD+ ratio and the Acetyl-CoA/CoA-ratio (Reed, 1981; Cardell et al., 1991). That may lead to an imbalance in activity regulation of PDH via phosphorylation and dephosphorylation, leading to an activation of pyruvate dehydrogenase kinase and, therefore, to a decreased activity of PDH by exceeded inhibition. It is unlikely, that the decrease of PDH in our study was due to a change in cofactor levels, as the used assay solution contained sufficient amounts of cofactors (such as pyruvate and NAD), although the total amounts of pyruvate and NAD were not measured in this study. Another supplement, that the decrease in PDH activity is due to the injury and not due to cofactor limitation is, that the brain samples were put into fluid nitrogen immediately and therefor NAD, a cofactor of PDH activity, had no time to degrade spontaneously (Balan et al., 2010).

On the other hand there are studies suggesting that oxidative stress is probably responsible for the loss of PDH activity, (Bogaert et al., 1994) as the production of superoxide, hydroxyl radical, nitric oxide, and peroxynitrite is elevated during reperfusion (Metodiewa and Koska, 2000). Summarizing these findings and the results of Liu et al. who found that hyperoxid resuscitation results in a more pronounced lipid peroxidation and worse neurological outcome, (Liu et al., 1998) it seems possible that the PDH is targeted by oxidative stress and that its inactivation may contribute to neuronal injury and neurologic impairment.

Lai et al. reported, that Ca^2+^ inactivates the PDH *in vitro* (Lai et al., 1988) and it was therefore concluded that an accumulation of Ca^2+^ in mitochondria could be responsible for the inactivation of PDH and for an imbalanced intracellular homeostasis of Ca^2+^ leading to a mitochondrial mediated cell death. However, Pandaya et al. showed recently a Ca^2+^-dependent inhibition of mitochondrial respiration without influencing PDH and NADH dehydrogenase (Complex I) (Pandya et al., 2013). With respect of the complexity of PDH regulation, even a combination of several mechanisms may be responsible for the reduction of PDH activity.

### Derangement of oxidative metabolism in (experimental and clinical) SAH

Derangement of metabolism and accumulation of metabolites after SAH have been observed in several studies (Sugi et al., 1975; Carpenter et al., 1991; Sarrafzadeh et al., 2001, 2005; Cesarini et al., 2002). MD studies in SAH patients revealed increased levels of lactate, pyruvate, lactate-pyruvate ratio (LPR), and glutamate, (Sarrafzadeh et al., 2001, 2005; Cesarini et al., 2002) measured on several points in time during SAH course. Information on metabolic changes in the early phase of SAH is rare, but Westermaier et al. discovered the same pattern of metabolites in the early phase of SAH in an experimental rat model, (Westermaier et al., 2011) suggesting a sustained metabolic depression after SAH. In addition, the incomplete recovery of CBF which exceeded the decline in ptiO_2_ seems to mirror the brain disability of O_2_-utilization observed in patient studies (Carpenter et al., 1991).

The dysfunction of cell metabolism is thought to start the cascade of ischemic cell death with an early brain edema, lactate acidosis, and free radicals, leading to a disturbed membrane potential, which results in an increase of extracellular glutamate and an influx of intracellular calcium. Cellular and mitochondrial Ca^2+^ overload, mitochondrial failure and the increase of excitatory amino acids are mediators of neuronal cell death. Finally this turns into a mismatch of supply and demand of energy. An elevated MD glucose concentration, glutamate, and LPR have been thought to correlate with poor outcome after SAH (Persson et al., 1996). The reduced activity of PDH shown in this study may lead to increased levels of glucose, lactate, pyruvate, and LPR, giving further support for an impaired aerobic glucose metabolism following SAH. A direct correlation of reduced PDH activity and deranged cerebral energy metabolism is still lacking. Indications for the hypothesis of a direct correlation could be the fact, that intravenous administration of acetyl-L-carnitine (ALCAR) reduced brain lactate levels and improved neurological outcome, (Rosenthal et al., 1992) probably due to the fact that the Acetylcarnitine-CoA transferase in brain allows ALCAR to enter the TCA cycle of neurons and astrocytes (Bresolin et al., 1982). The loss of PDH activity could lead to secondary brain damage due to secondary mitochondrial failure—as the failure of mitochondrial function after SAH has already been observed by Marzatico (Marzatico et al., 1998). Unfortunately it is not possible to answer the question, if and to what content the reduction in PDH activity correlates with cell death and therefore secondary brain damage following SAH in our study model, as we euthanized animals 3 h after SAH (to gain information on PDH activity at this particular and crucial time point of SAH course of disease) and the earliest time point to detect cell death is 24 h and would be even better in the following days after SAH. Therefore, further studies in our group have already been started addressing this question.

In summary, there might be a shift from aerobic to anaerobic metabolism already in the early phase after SAH, as we observed for the first time a reduced activity of PDH combined with a significant increase of lactate, LPR, and glutamate.

### Clinical implications

Our results indicate that there may be a primary enzymatic dysfunction of PDH—a key enzyme to the TCA-cycle—after SAH. Hence, the normalization of CBF in this crucial period may not be sufficient to prevent secondary brain damage, as the earliest establishes time-point of treatment of SAH-patients is 3 h after SAH, which is also the time-point when most patients who suffered severe SAH reach a hospital and have their diagnosis of SAH. If aerobic metabolism fails, the brain will eventually undergo secondary damage, typically selective neuronal damage as has repeatedly been found in this experimental model. It cannot be concluded from this data how big the impact of this metabolic derangement on brain damage is and how its long-term course is. However, there could be a potential target for a future neuroprotective treatment of SAH patients already in the early phase after SAH, immediately after hospital admission. Our data indicates also that the inactivation of PDH represents an independent pathophysiological factor, which might contribute to secondary brain damage after SAH. The protection against PDH inactivation as well as compensation for metabolic disruption could exert protective efficacy and act as a neuroprotective factor. Ca^2+^-channel blockers were found to have a beneficial effect on metabolic parameters, histological damage, and clinical outcome after cerebral ischemia in animals and humans, although the mechanism is still not fully understood. Dichloroacetate (DCA) was shown to stimulate PDH activity by inhibition of PDH kinase. The administration of DCA in animal models of global and focal ischemia led to a decrease of brain lactate levels and to an improvement of neurological outcome (Corbett et al., 1998) as well as to a decrease of brain lactate levels after administration in patients within the first 2 days after stroke (Graham et al., 2000).

Another target for a potential neuroprotective drug would be bypassing the PDH reaction and thereby provide “fuel” to the TCA cycle, which could be possible via ALCAR or ketone-bodies. Finally, if oxidative stress is responsible for the damage of the PDH, the treatment of radical formation may improve the PDH activity and exert a neuroprotective effect by this mechanism. All these drugs and treatments could be potential preventers of a PDH dysfunction but—with the exception of antioxidants—haven't been tested in the early phase of SAH until now.

## Conclusion

For the first time, this study shows a significant reduction of PDH activity in the early phase of experimental SAH in rats, which may play part in the derangement of oxidative metabolism found in previous studies. The inactivation of PDH seems to be an independent pathophysiological factor, which might contribute to secondary brain damage after SAH. Further studies will have to investigate, if PDH is the only affected enzyme or if there is more damage further “downstream” the aerobic glucose metabolism.

## Author contributions

All authors (NL, HF, CS, SK, RE, TW) contributed to the conception of the study and to acquisition, analysis, and interpretation of data. They were all drafting the work or revising it critically for important intellectual content. Final approval of the version to be published was performed by all authors. They all gave agreement to be accountable for all aspects of the work in ensuring that questions related to the accuracy or integrity of any part of the work are appropriately investigated and resolved.

## Funding

This publication was supported by the Open Access Publication Fund of the University of Würzburg.

### Conflict of interest statement

The authors declare that the research was conducted in the absence of any commercial or financial relationships that could be construed as a potential conflict of interest.
